# A Stanford Conference on Social Media, Ethics, and COVID-19 Misinformation (INFODEMIC): Qualitative Thematic Analysis

**DOI:** 10.2196/35707

**Published:** 2022-02-15

**Authors:** Michael A Gisondi, Daniel Chambers, Tatum Minh La, Alexa Ryan, Adyant Shankar, Athena Xue, Rachel Anne Barber

**Affiliations:** 1 Department of Emergency Medicine Stanford School of Medicine Palo Alto, CA United States; 2 Stanford University Palo Alto, CA United States

**Keywords:** COVID-19, infodemic, misinformation, disinformation, vaccine, social media, thematic analysis, qualitative

## Abstract

**Background:**

The COVID-19 pandemic continues to challenge the world’s population, with approximately 266 million cases and 5 million deaths to date. COVID-19 misinformation and disinformation led to vaccine hesitancy among the public, particularly in vulnerable communities, which persists today. Social media companies are attempting to curb the ongoing spread of an overwhelming amount of COVID-19 misinformation on their platforms. In response to this problem, the authors hosted *INFODEMIC: A Stanford Conference on Social Media and COVID-19 Misinformation* (INFODEMIC) to develop best practices for social media companies to mitigate online misinformation and disinformation.

**Objective:**

The primary aim of this study was to develop recommendations for social media companies to address the COVID-19 infodemic. We report the methods used to execute the INFODEMIC conference, conference attendee engagement and analytics, and a qualitative thematic analysis of the conference presentations. The primary study outcomes were the identified themes and corresponding recommendations.

**Methods:**

Using a constructivist paradigm, we conducted a thematic analysis of the 6-hour conference transcript to develop best practice recommendations. The INFODEMIC conference was the study intervention, the conference speakers were the study participants, and transcripts of their presentations were the data for this study. We followed the 6-step framework for thematic analysis described by Braun and Clarke. We also used descriptive statistics to report measures of conference engagement including registrations, viewership, post-conference asynchronous participation, and conference evaluations.

**Results:**

A total of 26 participants spoke at the virtual conference and represented a wide array of occupations, expertise, and countries of origin. From their remarks, we identified 18 response categories and 4 themes: trust, equity, social media practices, and interorganizational partnerships. From these, a total of 16 best practice recommendations were formulated for social media companies, health care organizations, and the general public. These recommendations focused on rebuilding trust in science and medicine among certain communities, redesigning social media platforms and algorithms to reduce the spread of misinformation, improving partnerships between key stakeholders, and educating the public to critically analyze online information. Of the 1090 conference registrants, 587 (53.9%) attended the live conference, and another 9996 individuals viewed or listened to the conference recordings asynchronously. Conference evaluations averaged 8.9 (best=10).

**Conclusions:**

Social media companies play a significant role in the COVID-19 infodemic and should adopt evidence-based measures to mitigate misinformation on their platforms.

## Introduction

The COVID-19 pandemic has taken a huge toll on the world. COVID-19 is currently the third leading cause of death after heart disease and cancer, with approximately 266 million cases and 5 million deaths to date [1,2]. More than 780,000 Americans have died from COVID-19, and new variants of the virus continue to emerge. However, after an initial year of staggering case numbers, we finally have observed a decrease in the number of new infections requiring hospitalization as more people get vaccinated against the virus [1]. As of December 2021, 60% of the US adult population had received 2 doses of the COVID-19 vaccine [3,4]. Still, this fell short of the US Centers for Disease Control and Prevention (CDC) goal of 70% vaccination of the US population by July 4, 2021 [5]. This disparity persists despite widespread vaccine access [3].

Throughout the COVID-19 pandemic, there has been a proliferation of both misinformation and disinformation about the virus, its origin, the vaccines, and potential treatments. Misinformation refers to inaccurate information disseminated without malice, while disinformation is the purposeful spread of inaccurate information with malicious intent (Table 1). Taken together, the escalation of inaccurate information surrounding the pandemic can be accurately described as the COVID-19 infodemic. According to the World Health Organization, an infodemic is “too much information including false or misleading information in digital and physical environments during a disease outbreak” [6]. The COVID-19 infodemic has significantly contributed to vaccine hesitancy, which is the refusal of vaccines when access is not a limiting factor [7], across the United States. In the case of COVID-19, this hesitancy occurs despite an excellent vaccine safety profile. COVID-19 vaccine hesitancy is higher in some demographic groups who have even lower vaccination rates than the general population, demonstrating that the infodemic may disproportionately affect some communities [8,9].

**Table 1:** List of key terms and definitions.

Key terms	Definitions
Misinformation	False or incorrect information that is spread without malice
Disinformation	Inaccurate information that is spread deliberately with a deceitful or harmful intent
Infodemic	Excess amount of information on a topic that usually spreads rapidly and is confusing or unreliable
Vaccine confidence	Belief that vaccines are effective and safe
Vaccine hesitancy	Delay in acceptance or refusal of vaccinations despite vaccine availability
Vaccine refusal	Refusal of all vaccines including childhood vaccines

Social media platforms accelerated the dissemination of inaccurate information about the pandemic, contributing greatly to the COVID-19 infodemic and its health effects [10]. Social media has been shown to be more effective at promoting vaccine hesitancy than uptake, leading to a reduced effectiveness of public health measures and to decreased public engagement in disease prevention activities [7]. This was made worse by influential political representatives and cultural figures who spread misinformation and disinformation across all major social media platforms, which is referred to as top-down misinformation [11]. Although the connection between vaccination rates and the presence of misinformation online is known, best practices for mitigating the COVID-19 infodemic are yet to be determined. Thus far, there are no uniform efforts and policies by social media companies to combat harmful misinformation and disinformation present on their platforms. Additionally, the effect of a coordinated effort by social media companies to act against the COVID-19 infodemic is untested.

In August 2021, we hosted *INFODEMIC: A Stanford Conference on Social Media and COVID-19 Misinformation* to address issues related to the ongoing COVID-19 infodemic. Our primary aim for the conference was to develop best practices for social media companies to mitigate the COVID-19 infodemic online. In this paper, we report (1) the methods used to execute the virtual conference, (2) conference attendee engagement and analytics, (3) our qualitative analysis of the conference presentations, and (4) best practice recommendations.

## Methods

### Study Design, Setting, and Population

We conducted a thematic analysis of the transcript of a 6-hour, virtual conference about the COVID-19 infodemic. Our study aim was to identify best practices for social media companies to combat COVID-19 misinformation online; results of our thematic analysis represent the primary outcomes of the study. The transcript of the conference presentations formed the data for this study.

The conference was called *INFODEMIC: A Stanford Conference on Social Media and COVID-19 Misinformation,* and it was sponsored by Stanford University (Stanford, CA) [12]. It occurred on August 26, 2021, and data analysis was completed between October 2021 and November 2021. The study participants each consented to have their presentations recorded. These videos are now in the public domain; thus, no further consent was sought for this analysis. The Institutional Review Board (IRB) of Stanford University (IRB# 63151) deemed this study exempt.

The complete video recording of “INFODEMIC: A Stanford Conference on Social Media and COVID-19 Misinformation” is provided in Multimedia Appendix 1. This represents the raw data used for our qualitative thematic analysis.

### Study Intervention

To meet our study aim, deliberate design and optimal execution of the conference were pivotal to the collection of meaningful data. We (RAB, MAG) convened 2 committees to assist with the project, one advisory and one for conference planning. First, we recruited a steering committee to oversee the project and provide strategic direction. The steering committee consisted of individuals from the following entities at Stanford University: the School of Medicine, the Internet Observatory, the Social Media Lab, the Digital Civil Society Lab, and the Health Communication Initiative. Members of the steering committee helped select and recruit some of the conference speakers, chose a date for the conference, and addressed day-of-conference logistics. Additionally, we recruited a planning committee (DC, TML, AR, AS, AX) that met weekly in the months leading up to the conference. The planning committee was comprised of Stanford students who successfully completed a course on the use of social media for knowledge translation in medicine. Students planned the conference under the direction of the steering committee and principal investigators.

We organized a conference agenda consisting of panel discussions and brief didactic presentations on a broad range of topics related to the COVID-19 infodemic and our study aim. We recruited expert speakers based on these topics from a variety of geographical, cultural, and academic backgrounds with special consideration of individuals who have power to influence vaccine-hesitant individuals. We used a diversity and inclusion lens to ensure a racial, ethnic, and gender balance of the participants in an attempt to mirror the general population who use social media platforms.

We created a conference website using Squarespace (Squarespace Inc, New York, NY) that served as the conference program, registration portal, and a platform to host 2 public calls for scholarly works [12]. The website and conference information were open-access, and conference registration was free. In addition to registration and advertising, the website promoted a call for research abstracts to be presented at the conference and a call for papers for a special theme issue of the *Journal of Medical Internet Research (JMIR)*: “Social Media, Ethics, and COVID-19 Misinformation.” We scored submitted abstracts using Google Form (Google, Mountain View, CA) and selected the best submissions for presentation during a research symposium that immediately followed the conference. The research symposium served as a means of crowdsourcing additional information that might inform our study aim and allowed for presentation of relevant new research in the field. JMIR guest editors reviewed manuscript submissions to the theme issue separate from our study team.

We advertised the conference to potential attendees through social media and email outreach. We created Twitter (Twitter, San Francisco, CA) and Instagram (Meta Platforms Inc, Menlo Park, CA) accounts 4 months prior to the conference. We posted educational content, speaker spotlights, and promotional information to these accounts biweekly, increasing in frequency as the conference date approached. These accounts were also used for same-day conference backchanneling. We encouraged the participants to publicize the conference on their social media accounts, and we promoted the conference through Stanford Healthcare email distribution lists and newsletters.

Given COVID-19 travel restrictions, we held the conference virtually and opened registration to the public. We selected Hopin (Hopin Ltd, London, England) as our virtual conference platform because it provided important features necessary for moderated panel discussions: multiple speakers on the screen at the same time, audience interaction through chat or microphone, polling, and question and answer functions. We selected August 26, 2021 for the conference date based on our steering committee’s accurate prediction of a COVID-19 vaccine surplus in the United States and the peak of news media attention about vaccine hesitancy. This date also minimized speaker conflicts and allowed enough time to plan the conference. INFODEMIC occurred 4 days after the US Food and Drug Administration formally approved the Pfizer (Pfizer Inc, New York, NY) COVID-19 vaccine, at a time of great public discourse over COVID-19 vaccine hesitancy.

On the day of the conference, we (RAB, MAG) met in person to manage conference logistics such as speaker login, backstage preparation, and online platform management. Virtually, other planning committee members (DC, TML, AR, AS, AX) oversaw the audience chat, monitored the questions and answers, took notes on key proceedings, and shared updates from the conference on social media. Abstract presentations followed the main conference program, as did a Stanford University student panel discussion on vaccine hesitancy led by the planning committee.

### Participant Sampling and Data Collection and Analysis

We used a combination of purposive and snowball sampling to identify a diverse participant cohort [13,14]. We first determined the topics of the participant panel discussions based on the study aim, desired flow of the conference agenda, and format (eg, number of sessions, topics, length). We then identified expert participants for each panel via internet searches, past publications, the organizations they represented, social media platform searches, word-of-mouth recommendations, and steering committee recommendations. Our a priori search criteria identified participants for 7 of the 10 scheduled presentations. The remaining 3 presentations had participants who were referred to us by these experts (these included panels of social media representatives, government or religious leaders, and ethicists). We digitally recorded the conference and used Zoom (Zoom Video Communications Inc, San Jose, CA) to transcribe the 10 individual presentations.

Using a constructivist paradigm, we performed a thematic analysis of these transcripts to identify best practice recommendations for addressing COVID-19 misinformation and disinformation online [15-17]. We followed the 6-step framework for thematic analysis described by Braun and Clarke [17]. We inductively coded the transcript data for the existence of concepts related to COVID-19 misinformation, not the frequency they appeared in the transcript [17,18]. We analyzed the transcripts to the level of sentences, grouped these responses into loose categories or concepts that were not predefined, and ignored irrelevant words [19]. Six study team members agreed on this preliminary coding approach and crafted rules before independently coding the content. We then met frequently to discuss code generation and meaning. Two study investigators were assigned to code each of the 10 transcripts separately, and these pairings were different for each transcript. Our final codebook consisted of codes agreed upon between the rater pairs. Using a consensus approach, we then conducted a team-based thematic analysis of the codes in a series of discussions among all of our investigators [15-17].

We used descriptive statistics to report measures of conference participation, engagement, and other analytics.

### Reflexivity

We acknowledge that the experiences and opinions of our study investigators may have influenced our data analysis in this constructivist paradigm. Our senior investigator (MAG) is an emergency physician and medical education researcher who teaches a course on social media in medicine called, “Does Social Media Make Better Physicians?” Our study team includes 6 Stanford University undergraduate students (RAB, DC, TML, AR, AS, AX) who each completed that social media course and a subsequent research course that facilitated this study. Because of their common experiences in these 2 courses, all of our study investigators may have shared a preconceived perspective and understanding of the COVID-19 infodemic; we explicitly examined this bias throughout the generation of codes and themes. The students were novices to qualitative research; however, the senior investigator has ample experience with the analytic approach used and oversaw each methodological step. All coding was done in pairs of undergraduate students to balance prior experiences and assumptions. We designated our senior investigator as a potential third reviewer for the few coding disagreements that required adjudication. We maintained a research diary during the study that recorded all coding sessions, group discussions, and decisions during analysis of the transcripts. We reflected on the experience of engaging in this study at each team meeting.

## Results

### Conference Participation, Engagement, and Analytics

#### Participants

We recruited 26 conference participants with diverse expertise and occupations (Table 2). The participants were assigned to speak individually or in panels based on their areas of expertise (Table 3). We selected 9 abstracts for presentation in the research symposium. The authors represented 8 different countries.

**Table 2:** Demographic characteristics of the conference participants (age, gender, and race were not collected from participants).

Occupation (n=26)	Results, n (%)^a^
Physician	10 (38)
Ethicist	4 (15)
Social media influencer	4 (15)
Social media company representative	3 (12)
Public health expert	3 (12)
Politician	1 (4)
Religious leader	1 (4)

^a^Column does not total 100% because some participants had 2 occupations.

**Table 3:** Summary of the INFODEMIC conference presentations and participants.

Presentation title	Speaker(s)	Affiliation(s)	Presentation summary
Welcome Address	Seema Yasmin, MD	Stanford University	As the COVID-19 pandemic continues through its second year, the need for a strong public health infrastructure remains critical to mitigating the spread of misinformation. Partnerships with public health institutions are more effective than dissociating from them.
COVID-19 Update	Yvonne Maldonado, MD	Stanford University	Five billion vaccines have been administered worldwide, with 350 million administered in the United States alone. However, reaching marginalized populations remains a challenge, and access through community organizations must be a priority.
Vaccine Confidence	Heidi Larson, PhD	London School of Hygiene & Tropical Health	Much attention has been given to the sheer amount of misinformation flooding the public, but more important is the dynamic, fast-moving nature of misinformation that undermines credible science. Trust, rumors, and receptivity have played a large role in the disparate impacts and levels of confidence in misinformation observed between different countries.
Vaccine Hesitancy	Agnes Binagwaho, MD, PhD, MA; Gloria Giraldo, PhD, MPH; Aida Habtezion, MD, MSc; Seema Yasmin, MD (moderator)	University of Global Health Equity (AB); Latino Health Access (GG); Pfizer Inc (AH); Stanford University (AH, SY)	More than vaccine hesitancy, issues about vaccine equity and access must be addressed, especially because global pandemic inequities have been so apparent. Trust is the best way to minimize vaccine hesitancy––trust in government officials, trust in physicians, and trust in the local community. This trust cannot be built overnight but must be built in a grassroots, day-to-day manner. Global vaccine access disparities are alarming because of mismanaged vaccine distribution in high-income countries, leaving low-income, at-risk nations vulnerable.
COVID-19 and Distrust of Healthcare	Italo Brown, MD, MPH	Stanford University	There are significant racial disparities in vaccine acceptance, especially among the Black and Latinx communities. Combating these disparities means “fighting the misinformation Olympics,” which refers to some communities having access to adequate information versus those who do not have credible information and trusted messengers. The embedded distrust of the health care system is an important etiology of vaccine hesitancy, and it must be contextualized in the history of communities of color, such as the devaluation of Black lives. There needs to be more communication of accurate information to communities of color, especially since information can often change and be distorted as it spreads. There also needs to be focus on restorative justice and validating mistrust; activating trusted messengers to enact change within communities and combat misinformation is essential.
Achieving COVID-19 Vaccine Equity	Tom Bollyky, JD; William A. Haseltine, PhD (moderator); Lisa Menning; Danielle Pacia, MBE	Council on Foreign Relations (TB); ACCESS Health International (WAH); World Health Organization (LM); The Hastings Center (DP)	A global approach to COVID-19 vaccinations is critical to ensure vaccine equity around the world. Thus far, vaccines have not been distributed to countries at highest risk, but instead 10 of the wealthiest countries are overrepresented in vaccine doses received. This is due to hoarding within wealthier countries, a lack of global cooperation and distribution, and a vaccine shortage worldwide. Knowledge sharing and infrastructure sharing are part of an important, related global disparity. In order to combat these issues, there needs to be greater transparency, more international cooperation, and delay of vaccine boosters until there is more universal coverage.
The Role of Social Media Companies	Aaron Berman; Brian Clarke; Renee DiResta (moderator); Anne Merritt, MD	Facebook (AB); Twitter (BC); Stanford Internet Observatory (RD); Google Search (AM); Stanford University (AM)	Twitter, Facebook, and Google each have policies for removing misleading content, adding warning labels, and deactivating accounts that promote misinformation. A major challenge for these companies is the removal of a huge influx of disinformation while promoting quickly evolving, high-quality, and medically accurate content. Collaborative efforts between social media companies and public health experts help fact-check and spread accurate information online. In the process of regulating online content, it is important for social media companies to balance free speech, censorship, and safety.
Leveraging Physician Influencers: The New Public Health Educators	Vin Gupta, MD, MPA	University of Washington; MSNBC Contributor	Social media platforms can be used effectively to tell stories and powerfully amplify accurate information, especially through visual media like videos and graphics. These platforms generate useful ideas, distribute actionable advice, build trust, and hold sources of misinformation accountable. Impactful, accurate medical information can be communicated online by leveraging multiple forms of media to engage different audience constituencies.
Do Social Media Influencers Affect Vaccination Rates?	Sanjay Juneja, MD; Jessica Malaty Rivera, MS; Cedric “Jamie” Rutland, MD	Baton Rouge General (SJ); Infectious Disease Expert at the COVID Tracking Project (JMR); West Lung (CR); Vice President of Association for Healthcare Social Media (CR)	Social media influencers can affect vaccination rates through effective science communication. Their messaging can make people confident about the vaccine, allow them to make choices out of a place of knowledge, and teach them to be discerners of truth. Empathy, respect, and relatability are crucial to empowering listeners to seek information, rather than antagonizing and shaming. Mitigating misinformation can come from debunking myths and anticipating logical fallacy traps people may espouse.
Role of Government and Religious Leaders in Mitigation Disinformation	Adrian Perkins; Gabriel Salguero, PhD; Matthew Strehlow, MD (moderator)	Mayor of Shreveport, Louisiana (AP); Reverend of the Gathering Place (GS); President of the National Latino Evangelical Coalition (GS); Stanford University (MS)	Combating COVID-19 disinformation relies heavily on collaborative partnerships. The partnership between public health and religious figures inspires trust and increases receptivity for the public, especially as trust in government officials declines. The politicization of the pandemic has contributed to distrust between opposing parties and threatened the mission of the faith community. Thus, spreading verified information and amplifying trusted voices within religious spaces are crucial to ensure that messages are communicated safely and reliably.
Ethical Imperatives for Social Media Companies and Influencers to Act	Nancy Berlinger, PhD (moderator); Arthur Caplan, PhD; Travis Rieder, PhD	The Hastings Center (NB); New York University (AC); Johns Hopkins University (TR); Berman Institute of Bioethics (TR)	Both effective science communication and ethical communication must be prioritized throughout the pandemic. The ethical imperatives of social media companies should include stricter self-regulation and refined presentations of information for the public. Social media companies are information disseminators, not journalists or reputable news people.

#### Same-Day Engagement

**Figure 1:**
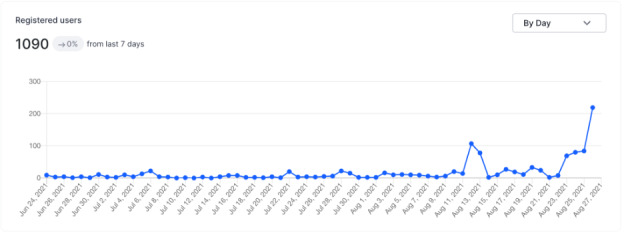
Most registrations occurred in the same week as the conference.

**Figure 2:**
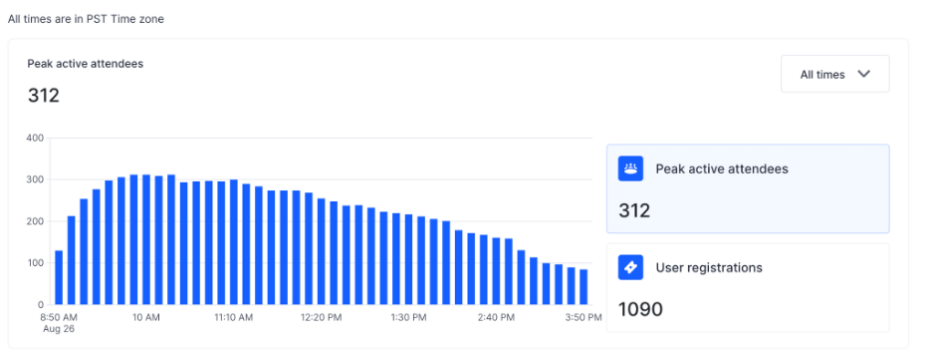
Peak active conference attendees by time.

A total of 1090 conference registrants came from 71 different countries, and 587 registrants (587/1090, 53.9%) viewed the meeting live. Most registrations occurred during the days leading up to the conference (Figure 1). Peak attendance was 312 viewers (312/587, 53.2%) at any one time, and the average viewing time was 2 hours 44 minutes (164/360 contiguous minutes, 45.6%; Figure 2). The mean rating by attendees of the conference quality was 8.9 (lowest: 0 to highest: 10). Table 4 summarizes the complete conference analytics.

**Table 4:** Conference analytics as of December 1, 2021.

Analytics			Results
**Registration analytics**			
	Registration fee	Free	
	Total registrations, n	1090	
	Final week registrations, n	486	
	Same-day registrations, n	219	
	International registrations, n	340	
	Countries with registrations, n	71	
**Top countries by registrations (n=1090), n (%)**			
	United States	747 (68.53)	
	Canada	39 (3.57)	
	Turkey	35 (3.21)	
	Philippines	23 (2.11)	
	United Kingdom	18 (1.65)	
	Other	228 (20.93)	
**Attendance analytics**			
	Total live attendees, n (% turnout)	587 (53.9)	
	Average attendee evaluation (out of 10; 10=high)	8.9	
	Peak attendance, n	312	
	Conference length (hours)	6	
	Average viewing time (minutes)	164	
**Asynchronous engagement, n**			
	Total YouTube viewers	929	
	Total Podbean listeners	9067	
	Instagram [20] followers	338	
	Twitter [21] followers	183	
	Unique website [12] views	5905	
**Conference links**			
	YouTube	[22]	
	Podbean	[23]	

#### Asynchronous Engagement

**Figure 3:**
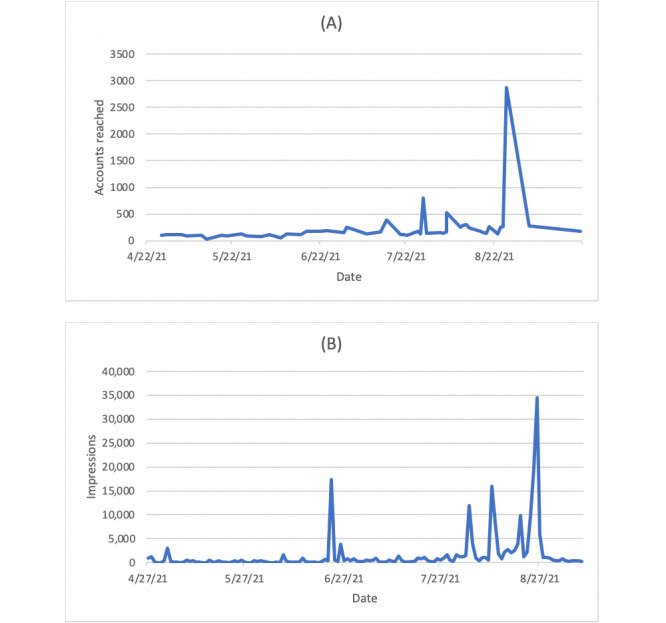
Engagement with our social media accounts by date: (A) unique Instagram accounts reached and (B) Twitter impressions.

**Figure 4:**
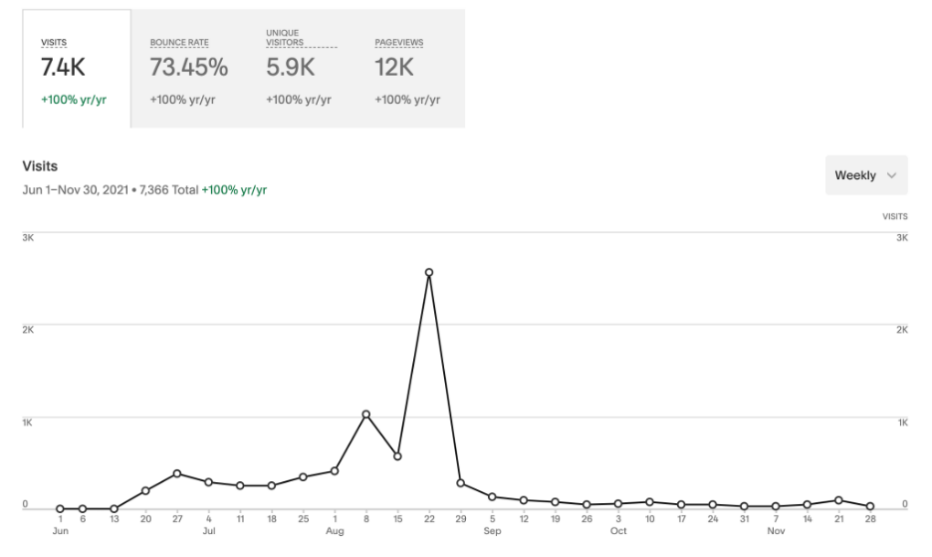
Website analytics showing a peak in visits during the week of the conference, August 22, 2021.

We distributed a complete recording of the conference to all registrants for asynchronous viewing, including the 503 registrants who did not view the live broadcast. Additionally, a recording of the conference and its individual presentations is available via YouTube, and each presentation was recorded as individual podcast episodes hosted by the *Academic Life in Emergency Medicine* Podcast via Podbean (New York, NY) [22-24]. This adds asynchronous INFODEMIC “attendees” totaling 929 viewers and 9067 listeners as of December 1, 2021. Our conference Twitter and Instagram accounts had a combined following of 521 users and reached tens of thousands of others (Figure 3). Our website had approximately 5900 unique visitors and 12,000 page views (Figure 4).

### Thematic Analysis

#### Overview

**Figure 5:**
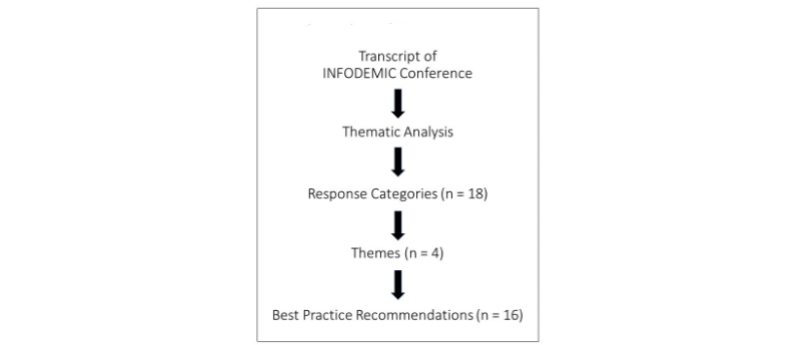
Overview of our data analysis.

The digital recordings of the 10 individual presentations resulted in 297 pages of transcribed text. Our analysis of this complete transcript resulted in 18 loosely associated categories of participant responses. These led to the identification of 4 major themes: trust, equity, social media practices, and interorganizational partnerships (Figure 5). These categories and themes broadly reflected the lack of access to both information and vaccines within marginalized populations, the global impact of COVID-19, and actions to minimize the spread of online misinformation (Table 5). Participants affirmed the ethical imperative on social media companies to use their platforms to curb the spread of COVID-19 misinformation because it continues to drive vaccine hesitancy and inequity.

**Table 5:** The 4 themes and 18 response categories identified in the data analysis.

Theme	Categories
Trust	Historical mistreatment of marginalized populations contributes to their present-day distrust of health care. Rumors, infodemics, and mistrust contribute to the spread of misinformation. Community-based outreach is important for building trust. Individual, collective, and commercial accountability is needed to reduce COVID-19 misinformation.
Equity	Inequitable access to accurate COVID-19 information fuels vaccine hesitancy and the disparate vaccination rates observed in some communities. Inequitable access to accurate information results from the lack of representation of persons of color in communications and media. COVID-19 vaccine access is more important than vaccine hesitancy globally. Health disparities are directly related to social determinants of health.
Social media practices	Strategic design of social media platforms can reduce misinformation. Empathy and respect are critical when communicating online with people who disagree with you. Social media involves managing the different values and interests of users. A balanced approach to free speech, censorship, and psychological safety is needed. Social media companies can educate users by flagging and removing misinformation and promoting correct information. Social media users can be taught how to critically analyze online information.
Interorganizational partnerships	Public health institutions should partner to combat the global impact of COVID-19. Community health initiatives can fight COVID-19 misinformation. Partnerships between social media companies and public health organizations can disseminate credible health information and fact-check online information. Social media platforms can connect the science community with governmental agencies, commercial brands, and local health organizations.

#### Trust

Trust was the fundamental issue in discussions of COVID-19 vaccine hesitancy throughout the conference. Our participants described how disparities in COVID-19 vaccine acceptance disproportionately affected marginalized populations due to the “historical lack of trust by some communities because they have been mistreated by science, mistreated by politicians, mistreated by the judiciary system, and are still mistreated by [law enforcement]...so they don’t trust.” One participant recounted numerous instances where members of the Black community were mistreated, emphasizing how “the abuse of Black bodies creates the bedrock for this [modern] distrust” and “[currently] we still see elements of mistreatment in the medical system. So, it would make complete sense to know that Black communities have a lasting and lingering distrust of health care.” Participants described how marginalized communities also suffer from a lack of “access to adequate information” because “having trusted messengers, people who can interpret messaging [and] can deliver adequate truthful information, is a commodity, [but] it is a rarity and it’s scarce in [marginalized] communities.”

One participant noted that “credible information and trusted voices have a viral nature, which [is] important in the context of the rampant mis- and disinformation” and that “a crucial factor that determines the spread of information is trust.” The influence of rumors that threaten trust within different countries was emphasized, as in “one country, [rumors] fizzle out and die, [while] in another country, it disrupts a program and [shuts] down a vaccination initiative. It all depends on the receptivity and fertile ground [within each country that] either amplifies or mitigates the spread.” Participants described “the process of building trust” through community-based organizations, which may not be an issue of “restoring trust but building trust with the public for the first time given long histories of exploitation.” Several participants underscored the importance of ensuring individual, collective, and commercial accountability when dealing with COVID-19 misinformation on social media.

#### Equity

Our participants described how the COVID-19 pandemic highlighted countless global inequities in health care infrastructure and access to information and care. One participant said, “nothing to do with this pandemic is equal, from mortality and morbidity rate[s], from who’s most susceptible to infection, [to] the inequitable access to vaccine.” Participants said that social determinants of health such as poverty, frontline jobs, crowded living spaces, crowded public transit, food deserts, and inadequate insurance coverage can “lead to higher risk for COVID disease but also serve to alienate and isolate people from good information and leave them vulnerable to local pockets of misinformation.” Another participant offered the example that, for pediatric deaths from COVID-19 around the world, “low- and middle-income countries have two and a half times the risk of deaths than high-income countries.” The participants discussed how the pandemic has revealed this “uneven playing field,” especially in denying equitable access to the vaccine among poorer countries. A participant cited a study done by the Africa CDC showing that 80% of people in African countries, 65% in the United States, and 60% in France are willing to get vaccinated, but “as of May 2021...only 2% of the African population has got access to the vaccine.” Our participants agreed that vaccine access is more important than vaccine hesitancy in much of the world. These inequities not only are global but also plague people within small communities, as one participant noted, “[sometimes] it’s only a 15-mile difference” between having and not having access to a continuous medical system.

In the same way that inequities cause access challenges to vaccines and health care services, one participant explained that there are also some communities that are “more vulnerable to being targeted by disinformation campaigns and are more susceptible to believing misinformation.” Our participants discussed how Pfizer focused on equitable access to vaccine information and clinical trials while developing their vaccine, describing that Pfizer put in the effort to “involve and ensure diverse populations are included.” In addition to including minority groups in clinical trials, one participant described how the Pfizer vaccine development process involved partnering with “trusted civil societies and NGO [nongovernmental organizations] like the NAACP [National Association for the Advancement of Colored People], the National Black Nurses Association, the National Hispanic Medical Association” to approach vaccine development in an equitable and “culturally sensitive way,” considering factors such as “removing barriers in enrollment like language.”

#### Social Media and Best Practices

Social media companies and influencers both play important roles in limiting misinformation online, as well as spreading accurate and credible content. Participants acknowledged that online platforms including Twitter, Facebook, and Google all have policies for removing inaccurate information, flagging misleading content, and deactivating accounts that promote misinformation. For example, one participant shared that Twitter “remove[s] content with the highest propensity of harm and content that may invoke deliberate conspiracy theories relating to COVID-19 and COVID-19 vaccines,” mitigating its spread. Social media companies have also been able to promote and amplify high-quality, medically accurate information via COVID-19 information pages and links labeling relevant posts. Through these efforts, Facebook has been able to “direct more than 2 billion people worldwide to expert health resources through [their] virus information center,” and Google developed search features to “provide direct access to information from health authorities.” Participants said that artificial intelligence systems can help “scale the impact” of misinformation and send it to “fact-checking partners around the world [that can] rate [the] content and reduce the distribution of it” if deemed harmful. Continual monitoring and tracking of online discussions allow companies to “have a better sense from a policy perspective where to start shifting [their] resources and where to start really understanding the patterns associated with it.” The companies are working to navigate the challenge of regulating their platforms by finding a balance between “enabling people to express themselves freely while protecting the safety of [their] user community.”

Health care influencers on social media can also impact vaccination rates by communicating information responsibly and effectively on their platforms. One way is by storytelling and giving actionable advice through a variety of media, whether that be “threads on Twitter, videos, [or] graphics”—all powerful ways of “leveraging the impact of multiple forms of media to their greatest extent” and increasing audience engagement. Influencers can use their platforms to build trust and generate ideas as ways to “transform people’s minds, to bring them out of fear and into confidence about the vaccine—everything from flu to even COVID-19 and HPV [human papillomavirus].” Participants agreed that influencers can also help increase science literacy and teach viewers to make choices “out of a place of knowledge” and “increase people’s ability to discern what is good data, what is bad data, and even how to read charts.” Although educating viewers, it is crucial for health influencers to express empathy as an “undertone of the way in which [they] communicate...and empower people to seek this information out.” Antagonizing and “shame-based motivation to seek information sends [people] to the darkest places of the internet” and is not as effective as coming from a place of understanding and respect.

#### Interorganizational Partnerships

The final theme that emerged from our conference was the dire need for interorganizational partnerships. From the first panel to the last, our participants emphasized the importance of partnering with like-minded organizations to improve public health and the information disseminated on social media. One participant advocated that health experts “should partner with public health and not dissociate ourselves from them because we need to build [the] public health infrastructure...[to] not only get through the pandemic, but all public health issues.” They asserted the need to utilize “existing community engagement structures, local community leaders, faith-based organizations, community groups, and health centers” to “disseminate transparent and comprehensive information” for public good. These sentiments were echoed throughout the conference. As one participant stated, “partnerships are essential [and National Latino Evangelical Coalition] partners with the CDC and the Office of Health and Human Services to have webinars in the target language” to eliminate distrust in the health care system among the Latino community.

Lastly, participants agreed that social media companies have a responsibility to partner with health organizations to ensure that their platforms are “[addressing] a wide range of misinformation and the associated harms...[while] amplifying authoritative health information.” One participant noted that Facebook is “partnering with other organizations to reach low vaccination rate communities such as campaigns featuring Black doctors or nurses or Spanish language campaigns” and “using that data that [they’re] collecting in partnerships with academic institutions to judge the impact [on the pandemic] over time.” Participants also discussed partnerships that Facebook maintains with health experts to “remove content that has been debunked as false and leading to physical harm” as well as partnerships with “the global network of more than 80 fact-checking organizations around the world in more than 60 languages” to identify misinformation about COVID-19 or the vaccine. Similarly, it was noted that Google has similar partnerships with “national public health authorities” to “display their content front and center” so users have “direct access” to it.

## Discussion

### Principal Findings

Health misinformation on social media poses a threat to public safety, especially in the midst of the COVID-19 pandemic. Our participants described how the increased use of social media during the pandemic paved the way for misinformation, conspiracy theories, and rumors to flourish, thus increasing fear and reluctance around the vaccine. With the emergence of new COVID-19 variants, it is especially vital that we combat vaccine hesitancy and vaccine inequity around the world. In particular, many of our participants acknowledged that social media companies have an ethical obligation to act against the spread of harmful misinformation on their platforms. Presently, social media companies are making efforts to mitigate misinformation—however, more could be done.

After analyzing transcripts from the INFODEMIC conference, we recommend 9 ways in which social media companies can better combat COVID-19 misinformation online (Table 6). While our intended goal was to make recommendations about COVID-19 misinformation to social media companies alone, our data were so rich that our analysis led to recommendations for health care professionals and the general public as well. During our analysis, we also identified several response categories specific to health care professionals and the general public, so we generated additional recommendations for each of these respective groups. Our findings are especially timely and important, as the literature has relatively few evidence-based guidelines for how social media companies should effectively manage the COVID-19 infodemic [25-27]. In addition, many health care professionals and social media users are unaware of the potential benefits of social media for the dissemination of factual scientific information and the promotion of COVID-19 vaccines [25,28,29].

**Table 6:** List of recommendations for social media companies, health care professionals, and the general public to mitigate COVID-19 misinformation.

Target	Recommendations
Social media companies	Increase representation by people of color as messengers of factual information to build trust in medicine and science within marginalized communities. Promote posts from trusted sources of credible information. Fact-check, flag, and remove posts that propagate the spread of misinformation. Adjust search engines and other algorithms that push misinformation. Use easily understandable information, infographics, and messaging. Remind users to be critical of information they read online. Partner with public health organizations to spread credible information. Partner with one another in a coordinated effort to combat COVID-19 misinformation. Encourage vaccinations and equitable access to vaccines.
Health care professionals	Engage in public health education online. Use social media platforms to connect scientists, government officials, and health care organizations with the general public. Be mindful of global health disparities when messaging about the vaccines.
General public	Critically analyze social media content related to COVID-19. Disseminate scientific facts and evidence-based information. Search for information rather than rely on social media algorithms to push content to you. Be patient and empathetic in conversations with vaccine-hesitant individuals.

Some pre-COVID literature addressed health misinformation on social media platforms. Although these papers predate the current COVID-19 infodemic, our data support their overarching themes and recommendations. Chou et al [29] recommended a strong clinician-patient relationship for optimal health communication, and our findings underscore this importance of building patient trust in science, medicine, and health information messengers. Similarly, we found that individual and commercial accountability is needed to reduce COVID-19 misinformation. Chou et al [29] also recommended that social media companies implement mechanisms to validate information on their platforms. Our analysis yielded more detailed recommendations for social media companies to (1) flag and remove posts that propagate the spread of misinformation, (2) promote posts from trusted sources such as easily understood information and infographics, and (3) promote reminders to the public to be critical of what they see online [29]. Finally, Walter et al [25] performed a meta-analysis of pre-COVID studies that found that the most effective messenger of health information is a recognized expert in a given field, which aligns with the findings in our study as well.

Several unique aspects of the INFODEMIC conference planning and this subsequent study are noteworthy. The INFODEMIC planning committee and this author team were comprised primarily of undergraduate students (DC, TML, AR, AS, AX) who executed the event and this analysis as class projects. The students learned how to effectively work as a team, communicate with professional speakers, plan the logistics of a conference, operate the digital conference platform, and perform a qualitative analysis of conference transcripts. Second, our speaker selection was carefully planned so that a diverse group of fields was represented. For instance, we thought it was important to include religious leaders and physicians in discussions of social media regulations, a space normally dominated by social media companies and the government. The broad diversity of expert speakers was intentional, and this qualitative analysis was meant to discover commonalities among their approaches to health misinformation. Finally, we believe that our thematic analysis of a full conference transcript is uncommon in the literature and represents a rigorous method of summarizing knowledge generated at a scientific meeting.

Lastly, our experiences hosting an online conference are instructive. The virtual platform allowed us to convene a panel of experts from locations in North America, Africa, and Europe simultaneously. It is unlikely that we could have recruited the same panel to a live conference due to costs, travel during a pandemic, and schedule availability. Conference registrants similarly represented an international audience. We also purposefully recorded the conference and made it freely accessible online through YouTube videos and Podbean podcasts. This ensures that the conference has continued impact, as evidenced by the 10-fold increase in asynchronous, on-demand viewers to date compared with live conference attendees. Additionally, registrations peaked in the final 48 hours prior to the conference, an unanticipated finding that is anecdotally common for virtual conferences. This may have been due to simultaneously higher social media activity about the conference in the days prior, both from the INFODEMIC planning committee and the invited speakers. These experiences are consistent with expert guidelines for planning virtual meetings [30,31].

### Limitations

We acknowledge several important limitations of our qualitative study design. Most notably, our sampling technique is subject to selection bias, and thus, our data were contingent on the specific participants or panel discussion topics chosen. However, it is unclear if similarly selected content experts would have offered substantively divergent perspectives. We tried to address this issue by creating a diverse conference agenda, recruiting participants with content expertise, and performing a thematic analysis across all 10 of the conference presentations to increase the variety of participants’ responses in the data set. Two notably absent participant types were vaccine-hesitant individuals and “antivaccine” individuals. Though we initially intended to include a vaccine-hesitant participant, we eventually chose not to recruit any because of the difficulty of ensuring their psychological safety during the conference. Additionally, we were concerned that an “antivax” (vaccine-refusing rather than vaccine-hesitant) participant might have led the conference astray from its primary objective. Such individuals may have interacted with our participants during their presentations anyway, using the virtual conference platform chat function; this is not clearly reflected in our data set as the transcripts captured audio-only content, not chat room discussions.

Additionally, several biases reflective of participant comfort during the live, internationally broadcast conference may have affected the results; these include social desirability bias, acquiescence bias, and sponsor bias. Each of these response biases were partially mitigated by the use of open-ended questions and panel moderation by participants rather than sponsors. Similarly, status quo bias may have affected the responses of participants representing social media companies. Some responses may have been further biased by the use of a virtual conference platform to host the conference. Although this format allows for a global audience, technology itself can be a barrier for participants and alter their engagement and responses. Moreover, the conference was held in English without closed captioning or translation services, which may have affected attendee interaction with the participants via the chat function. Finally, our results may be subject to confirmation bias and to the experiential bias of the co-investigators who planned the conference and wanted to ensure its success. These were addressed by the use of paired investigators to code data and through direct supervision and instruction by the first author who has expertise in this methodology.

### Conclusions

The Stanford INFODEMIC conference brought together experts from various fields and backgrounds to discuss the etiologies of vaccine hesitancy and the power of social media to increase COVID-19 vaccination rates. There were common themes to the participant remarks: improving trust in science and medicine, promoting equity in vaccine access and health care, identifying social media best practices, and creating interorganizational partnerships. As the future course of the pandemic continues to be uncertain, we offered specific recommendations that social media companies, health care professionals, and the general public can adopt to better mitigate online health misinformation.

## Appendix

Multimedia Appendix 1 Stanford Infodemic Conference Recording.

## Abbreviations

CDC: Center for Disease Control and Prevention
HPV: human papillomavirus
IRB: institutional review board
JMIR: Journal of Medical Internet Research
NAACP: National Association for the Advancement of Colored People
NGO: nongovernmental organizations
