# Supplementary material for: A Stanford Conference on Social Media, Ethics, and COVID-19 Misinformation (INFODEMIC): Qualitative Thematic Analysis
Source: J Med Internet Res. 2022 Feb 15;24(2):e35707. doi: 10.2196/35707 (PMC8849255; doi:10.2196/35707)
Supplement: Multimedia Appendix 1 [file jmir_v24i2e35707_app1.doc]

**Multimedia Appendix 1: Stanford Infodemic Conference Recording**

Please find the link to the conference here: <https://www.youtube.com/watch?v=gYTt8pRB5d0&t=18635s>
